# Life on the edge: Mesolithic population size and viability on Malta

**DOI:** 10.1093/pnasnexus/pgag234

**Published:** 2026-07-28

**Authors:** James Blinkhorn, Lucy Timbrell, Matt Grove, Andrea Manica, Mario Mata-González, Huw S Groucutt, Nicholas C Vella, Eleanor M L Scerri

**Affiliations:** Department of Archaeology, Classics and Egyptology, University of Liverpool, 12-14 Abercromby Square, Liverpool L69 7WZ, Merseyside, United Kingdom; Human Palaeosystems Group, Max Planck Institute of Geoanthropology, Kahlaische Str. 10, 07745 Jena, Thuringia, Germany; Department of Archaeology, Classics and Egyptology, University of Liverpool, 12-14 Abercromby Square, Liverpool L69 7WZ, Merseyside, United Kingdom; Human Palaeosystems Group, Max Planck Institute of Geoanthropology, Kahlaische Str. 10, 07745 Jena, Thuringia, Germany; Department of Archaeology, Classics and Egyptology, University of Liverpool, 12-14 Abercromby Square, Liverpool L69 7WZ, Merseyside, United Kingdom; Department of Ecology, University of Cambridge, Downing St., Cambridge CB2 3EJ, Cambridgeshire, United Kingdom; Human Palaeosystems Group, Max Planck Institute of Geoanthropology, Kahlaische Str. 10, 07745 Jena, Thuringia, Germany; Department of Classics and Archaeology, University of Malta, Msida, Reġjun Lvant, MSD 2080, Malta; Department of Classics and Archaeology, University of Malta, Msida, Reġjun Lvant, MSD 2080, Malta; Department of Prehistoric Archaeology, University of Cologne, Kerpener Str. 30, 50937 Lindenthal, Germany; Department of Classics and Archaeology, University of Malta, Msida, Reġjun Lvant, MSD 2080, Malta; Human Palaeosystems Group, Max Planck Institute of Geoanthropology, Kahlaische Str. 10, 07745 Jena, Thuringia, Germany; Department of Classics and Archaeology, University of Malta, Msida, Reġjun Lvant, MSD 2080, Malta; Department of Prehistoric Archaeology, University of Cologne, Kerpener Str. 30, 50937 Lindenthal, Germany

**Keywords:** mesolithic, seafaring, Mediterranean prehistory, hunter-gatherer populations

## Abstract

Recent discoveries in Malta and Tunisia provocatively point to a possible web of Mesolithic/Epipalaeolithic sea crossings, encounters, and exchanges that linked continents long before the age of the sail. Determining whether such connections were singular events or sustained episodes is therefore critical to illuminate the scale and character of early seafaring. Here, we model how patterns of sea-level change impacted land connectivity between Sicily and Malta and predict potential population sizes through time from the Last Glacial Maximum to evaluate whether Mesolithic hunter-gatherers could have persisted on Malta, or if sustained connections would have been required. To achieve this, we use reconstructions of sea-level change and modern bathymetric models to evaluate changes in land size areas and connectivity. We calculate net primary productivity values for a high-resolution paleoclimate dataset, which we translate to population density by comparisons to ethnographic datasets. Finally, we calculate changes in estimated population sizes through time for Malta and Sicily, as well as minimum distances of sea voyages. Our results show that following disconnection from Sicily, Malta would have been unable to support a viable, persistent forager population, including for the minimum 1000-year timespan of the Maltese Mesolithic record. As a result, only repeated, long-distance sea voyaging can explain the longevity of Mesolithic occupations of Malta, opening up the possibility that other south-central Mediterranean islands were reached and explaining the presence of European hunter-gatherer ancestry in North Africa.

Significance Statement This study provides the first quantitative assessment of the long-term viability of Mesolithic forager populations on Malta, demonstrating that they could not have persisted over the long-term without continued population contacts with adjacent landmasses. By modeling changing human population size over time, we show that Malta's insular ecology could not have supported a self-sustaining hunter-gatherer population for the millennium in which they are so far documented. This finding implies repeated, long-distance sea crossings during the terminal Pleistocene and early Holocene, strengthening emerging evidence for a wider network of early maritime mobility linking Sicily, Malta, and North Africa long before the advent of sailing technology.

## Introduction

Human colonization of small and remote Mediterranean islands has often been thought to have been beyond the capabilities or aspirations of hunter-gatherer populations (1–3). Beyond the practicalities of reaching such locations, and the desire to do so, the fundamental issue becomes whether a viable population could be sustained after arrival. Island environments impose stronger constraints than those of continental settings: limited land area, finite and often fragile resource bases, low biodiversity, and demographic pressures arising from restricted carrying capacities. These challenges are both environmental (climatic/ecological) and social, as small, isolated groups risk demographic instability, as well as reduced genetic and cultural diversity (4–6). These challenges increase with decreasing land area and distance from the mainland and/or other islands. For this reason, islands serve not only as distinctive geographical case studies, but also as broader models for understanding contact/isolation thresholds, landscape use, and impacts on forager lifeways, since their physical boundaries make the ecological and demographic constraints on population dynamics more visible.

Research on forager population densities has often focused on the relationship between subsistence potential and environmental productivity, with net primary productivity (NPP) frequently used as a proxy for carrying capacity (7–13). This is because hunter-gatherers rely directly on resources available in their surroundings, which vary in abundance and energy content, and their survival and reproductive success are closely tied to the productivity of the environment. In this way, NPP provides a useful metric for estimating the demographic limits of forager populations across different ecological contexts. However, the amount of available food may not be all that matters in controlling hunter-gatherer abundance, with additional factors also shaping long-term viability, including demographic profile, exposure to disease, spatiotemporal variability in resource distribution, biodiversity, and the genetic and cultural risks of small population sizes (4, 8–10). It has been proposed that a meta-population of several hundred, if not over 1000, people are required for long-term forager survival (7, 14). In the case of island environments, sea-level fluctuations (11) and the capacity to supplement terrestrial productivity with marine resources (15) are likely to also be critical factors constraining the occupation potential of islands, yet the extent to which these resources could offset demographic and ecological constraints remains unresolved.

As well as having genetic implications, small population sizes carry profound cultural consequences. Mathematical modeling and experimental studies have shown that innovation rates and “cultural complexity” are closely tied to population size and interconnectivity (6, 16–18). On small islands, where contact with external groups could be sporadic or absent, the potential for cultural loss would have been heightened (6). The case of Tasmania is often highlighted in this regard: after sea level rise around 10 thousand years ago (ka) cut the island off from mainland Australia, the small population of Indigenous Tasmanians became isolated for millennia. Archaeological evidence suggests that over time, certain technologies and practices were lost, including fishing, the production of bone tools, and more complex stone tool technologies (5, 6). This example demonstrates how demographic isolation could drive cultural loss, raising important questions not only about the biological viability of early island populations but also about the long-term sustainability of their cultural repertoires. In particular, seafaring technology has been considered among the most complex aspects of hunter-gatherer culture (19, 20), implying that small population sizes may have had particularly significant impacts on island communities and the retention of such seagoing knowledge. Thus, continuity of voyaging may have been necessary for both demographic and cultural reasons (14, 20, 21).

Mediterranean islands provide an important arena for exploring population dynamics in the context of sea level change and climatic oscillations. The conventional view has long held that many of the smaller islands were first colonized in the Neolithic, since agriculture was assumed to be necessary to sustain permanent populations in such constrained environments with low productivity, low biodiversity, and unbalanced and impoverished faunas (3, 22, 23). However, recent archaeological evidence from Malta challenges this narrative. Traditionally considered to have been first settled in the Neolithic (24), recent research now shows that Mesolithic hunter-gatherers were present over at least a one-thousand year timeframe (25). This raises fundamental questions about the capacity of these forager populations to establish themselves on small Mediterranean islands. Was seafaring sustained over this timeframe, or was a single founding population able to survive, in isolation, for over a millennium, perhaps following an accidental arrival? If Malta was connected to Sicily during the terminal Pleistocene, is it instead possible that a stranded hunter-gatherer community was able to persist for several thousand years? If Mesolithic hunter-gatherers were able to sustain viable populations on Malta, then similar strategies may have been employed on other small islands across the Mediterranean. Such a scenario would not only reshape our understanding of the ecosystems and prehistory of these islands but would also challenge the assumption that agricultural economies were a prerequisite for successful long-term small-island colonization.

This study addresses these questions by modeling the demographic and subsistence potential of terminal Pleistocene to Holocene Malta and Sicily. Specifically, it explores how during the terminal Pleistocene and early Holocene shifting sea levels altered both the land area and the accessibility of these islands, while also examining how fluctuations in NPP and proximity to the coast impacted carrying capacity per unit area. Combining these effects permits analysis of the scale and duration of forager occupations that could have been supported. While extrapolating past forager densities from recent ethnographic datasets has inherent limitations, a range of research has shown that, with suitable subsetting of ethnographic calibration data, effective estimates of past forager densities can be obtained (26–29). Moreover, this approach builds the first testable framework for future, multidisciplinary evaluation. By situating Malta within this broader ecological and temporal framework, this study contributes to wider debates on the feasibility of small-island colonization by Mesolithic hunter-gatherers of the Mediterranean, the conditions under which small-island occupations could be sustained, and helps to illuminate aspects of the demographic, social, and technological characteristics of central Mediterranean populations prior to the arrival of farming.

## Results

**Figure 1:**
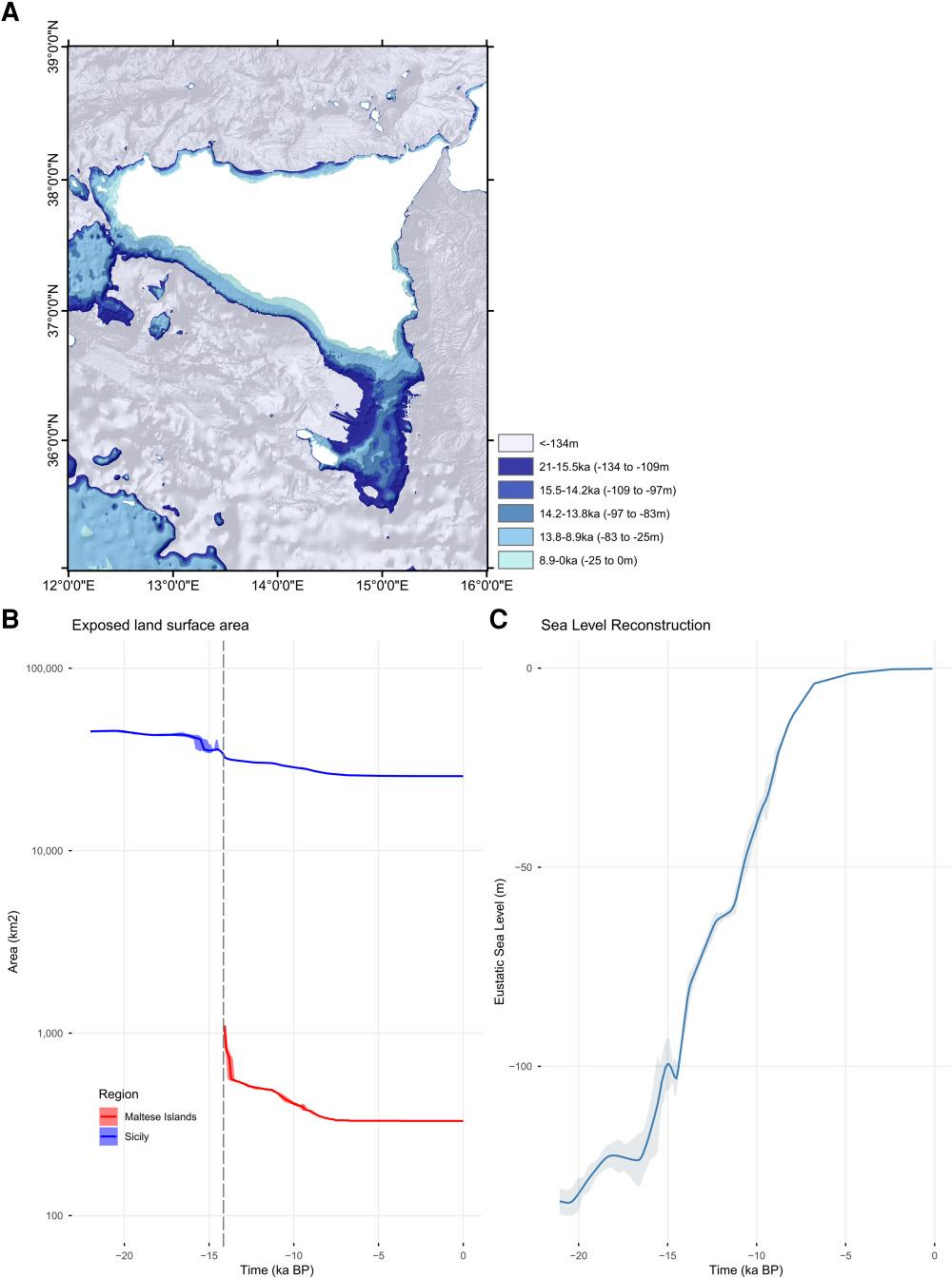
A) Map of bathymetry (GEBCO 2019 (22)) of Malta and Sicily highlighting phases of inundation resulting from changes in sea level (following Lambeck et al. (23)) leading to substantial decreases in Malta's land surface area (produced using ArcMAP 10.5); graphs illustrating B) land surface area for the Maltese Islands and Sicily and C) sea level change through time (23), based on average (line) with 2 SD (shaded ribbon) sea levels (following Lambeck et al. 2014); dashed line on (b) identifies the isolation of Malta from Sicily.

Combining models of modern bathymetry (30) and sea-level change (31) provides a means to examine changing connectivity between Malta and Sicily and fluctuations in land area during the terminal Pleistocene and Holocene (see Data and methods). Figure 1 illustrates how changing sea levels have resulted in substantial reductions in land surface area for Malta and Sicily from the Last Glacial Maximum (LGM, ca. 26–19 ka) to present conditions. Between the LGM and ca. 15.5 ka, Malta and Sicily formed a single large island (ranging between 45 and 40 thousand km^2^) with extensive terrestrial connectivity. Under these models, substantial reduction in land area occurred between ca. 15.5 and 14.2 ka, reducing the extent of terrestrial connectivity between Malta and Sicily, and reducing the combined landmass to 38–34 thousand km^2^. After ca. 14.2 ka, under these models the land-bridge between Malta and Sicily became fully submerged and the land surface area of Sicily decreased steadily, reaching 30 thousand km^2^ by ca. 11.2 ka, and settling at around 26 thousand km^2^ by ca. 8 ka. After disconnection from Sicily, the Maltese islands saw a rapid decrease in land surface area from 1.1 thousand km^2^ at ca. 14.1 ka to 561 km^2^ by 13.7 ka, with a gradual decrease to 369 km^2^ by 8.9 ka, shortly before the first documented arrival of humans in Malta (25). After this time, the Maltese islands of Malta, Comino, and Gozo became separated (together covering 316 km^2^), with Malta as the main island reaching its current size of 252 km^2^.

**Figure 2:**
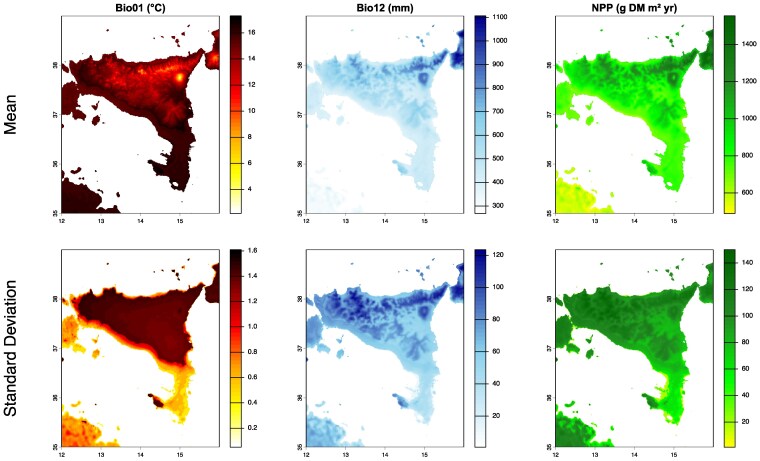
Maps illustrating mean (top) and SD (bottom) of MAT (Bio01) (left), TAP (Bio12) (center), and NPP (right), spanning the past 21 thousand years, with descriptive statistics calculated only for timeslices in which cells are predicted to be above sea levels for the time slice.

Alongside changes in land surface areas, substantial climatic change has occurred over the past 21 thousand years. Figure 2 illustrates averages for Mean Annual Temperature (MAT; Bio01) and Total Annual Precipitation (TAP; Bio12) evident in the CHELSA TraCE 21k dataset (32) for the study region (see Data and methods). The more varied topography of Sicily leads to a greater diversity in average values for Bio01 and Bio12 when compared to Malta. Past land surface areas that are now submerged present typically lower values of Bio01 and Bio12 as well as lower SD than Malta or the adjacent south-east coast of Sicily, reflecting both the short time frames of exposure as well as this occurring during periods where broader climatic patterns led to suppressed temperatures and increased aridity.

We applied the Miami model (33) to the temperature and precipitation values for the Maltese islands and most of Sicily from the CHELSA TraCE 21k simulations in order to calculate potential terrestrial NPP for the study area spanning the past 21 thousand years (Fig. 2; see Data and methods). The Miami model is a simple vegetation model in which productivity is controlled by limits of precipitation and temperature. NPP cell values across the study area and time frame range from 371 to 1794 g/m^2^/year, and NPP is highest, but more variable, in northern Sicily. To explore patterns of past population density, we examined the relationship between contemporary forager population densities (26, 34) with Miami NPP values for the modern (0 ka) timeslice of the CHELSA TraCE 21k dataset and the distance to modern coastline using generalized additive models (GAMs), with the distance to modern coastline offering a potential proxy for access to maritime resources.

We subset contemporary forager populations employed in modeling in two ways. Firstly, we divided analysis between foragers for which Miami NPP is controlled by either precipitation or temperature (P&T) from those controlled only by precipitation (P), the latter best matching the region of analysis. Secondly, we created subsets of both the P&T and P datasets to span the full scope of the ethnographic data, as well as with constraints on the proportional role of fishing (and other aquatic resources, referred to hereafter as “fishing”) in the diet, with thresholds at <50, <25, and <10%, offering another index for the influence of maritime resources on population size. We applied each model to past time slices of the Miami NPP dataset, factoring in changing land surface areas and impacts on distances to coastlines, to produce a model of population density (number of people per km^2^) through time (Fig. 2).

**Figure 3:**
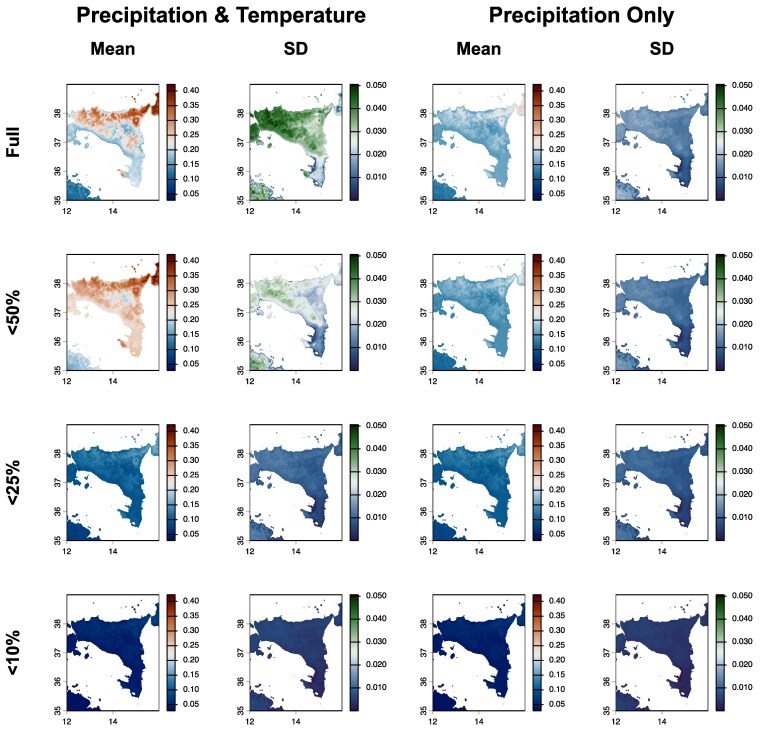
Maps illustrating the mean and SD of predicted population density derived from models based on alternate subsets of ethnographic datasets, including where Miami NPP is controlled by either precipitation and temperature (left) or only precipitation (right), on with different proportions of fishing included in the diet, from >50% (top) to <10% (bottom) spanning the past 21 thousand years, with descriptive statistics calculated only for timeslices in which cells are predicted to be above sea levels for the time slice.

Larger population density estimates were produced using ethnographic data from populations where Miami NPP estimates were derived from both precipitation and temperature limited habitats (P&T) rather than just precipitation (P). Broadly comparable population density estimates were produced from use of the Full and <50% fishing datasets, with substantial decreases observed with lower thresholds on the contribution of fishing to the diet. Below we report results based on highest overall density estimates (P&T < 50% fishing; deviance explained = 57.5%; *r*^2^ = 0.566) and the largest estimates from the precipitation-controlled dataset (P Full; deviance explained = 45.5%, *r*^2^ = 0.449). Across the study area and time frame, predicted forager population densities range from 0.469 to 0.107 per km^2^ and 0.282 to 0.086 per km^2^ for P&T < 50% fishing and P Full, respectively, with higher population densities predicted for northern Sicily, reflecting patterns observed in NPP values (Fig. 3).

**Figure 4:**
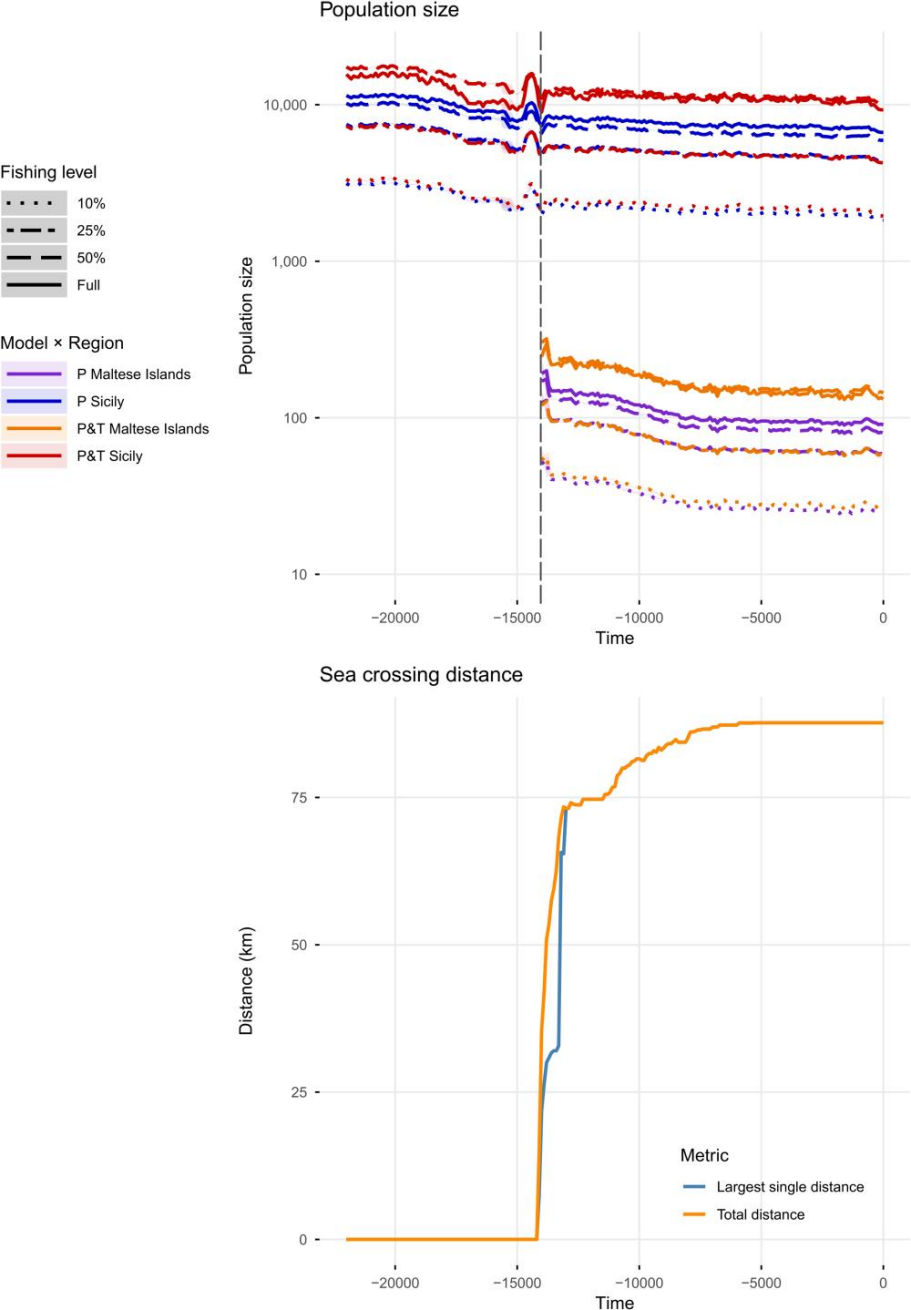
Predicted forager population size (top) for Sicily (red [P&T]; blue [P]) and the Maltese Islands (orange [P&T]; purple [P]) through time based on alternate models of the relationship between forager density with Miami NPP and distance to the coast, with varying thresholds on contributions of fishing to the diet, with the dashed gray vertical line identifying the isolation of Malta from Sicily, and (bottom) the total (yellow) and longest leg (blue) of the shortest sea crossing required between them.

To explore the potential viability of long-term occupations of Malta, we summed population densities, calculated as individuals/km^2^, by discrete landmass, in ca. 0.7 km^2^ cells, for each time slice to calculate the predicted forager population size (see Data and methods). We examine patterning in the predicted forager population size for Malta and Sicily through time (Fig. 4; results in the text are reported as P&T < 50% fishing/P Full). Until 14.2 ka, Malta and Sicily formed part of the same discrete landmass and shared patterning in predicted forager population size from 21 ka up to this point. Between 21 and 19.2 ka, predicted population size for the shared landmass of Sicily, Malta and exposed land surfaces range between ca. 16,767–17,735 (P&T < 50% fishing)/11,053–11,629 (P Full) individuals (following results are similarly presented as P&T < 50% fishing/P Full). Following this, a decline in predicted forager population size occurred, stabilizing between ca. 11,713–13,712/7,825–9,144 individuals between 17.1 and 14.9 ka. This is followed by a sharp increase reaching a maximum of ca. 15,834/10,30,000 individuals at 14.5 ka, contemporaneous with the Bølling–Allerød interstadial. As predicted, population size dropped sharply from this peak as Malta became isolated from Sicily. In Sicily, predicted population size dropped to ca. 10,964/7,324 individuals by 14.1 ka, before rising to between 10,691–12,945/6,963–8,498 individuals from 13.9 ka to 0.6 ka, after which predicted population size drops down to ca. 10,184/6,661 individuals for the present day. Following disconnection from Sicily, predicted population size for the Maltese islands and contiguous land areas dropped from ca. 419/265 individuals at 14.1 to ca, 319/199 by 13.8 ka, and gradually decreasing to ca. 206/127 by 10.5 ka. After 10.5 ka predicted forager population size decreases to ca. 170/106 individuals by 9 ka, then varied between 144–170/90–106 individuals until the present day.

To understand the relationship between Malta and Sicily following their disconnection, we conducted cost-path distance analyses to identify the total and largest single sea crossing required to travel from one to the other. In the absence of evidence for sea currents and how they may influence either the feasibility of the route or the energetic costs for making these crossing, these present only minimum estimates of sea-crossings required, based on straight line distances. Between 14.1 ka, directly after disconnection from Sicily, and 13.3 ka, a series of islands occurred between these landmasses, which reduced the longest individual crossing between them, typically halving the total crossing distance (Fig. 4). During this time frame the longest individual crossing increased from 7.5 to 33 km, in contrast to total sea crossings of 12.5 to 64 km. After 13.3 ka, the role of islands offsetting the sea crossing distance becomes negligible, with total crossing distances reaching 80 km by 10 ka, 86 km by 8.5 ka, and slowly rising thereafter to 88 km by 5.9 ka.

## Discussion

We combined high-resolution climate models of the past 21 thousand years with modern bathymetric data and sea level estimates to explore the impacts of Mediterranean climatic and sea level changes on population size over a region encompassing Malta and Sicily, including now-submerged landscapes. Building on Miami model estimates for NPP and changing distances to the coastline, we parameterize the relationship between forager population densities in the present, and apply this to the past, enabling us to estimate potential forager population sizes for Malta. Our results indicate that as Malta separated from Sicily after ca. 14.2 ka the crossing distance between these landmasses increased rapidly, reaching a minimum of 80 km within less than a millennium, during which time the surface area of Malta shrank considerably. Predicted maximum population sizes for Malta following disconnection from Sicily drop from a maximum of 419 individuals to 170 individuals by 9 ka, stabilizing between 170 and 150 individuals thereafter. Lower population sizes are predicted from models derived from ethnographic data where Miami NPP is precipitation-controlled, which is comparable to Malta and much of Sicily, as well as with decreasing contributions of fishing to the diet. Below, we discuss these results with particular focus on the recent discovery of forager occupations of Malta.

The Maltese Mesolithic, between ca. 8.5–7.5 ka (25), occurred when the surface area of Malta was comparable with its current state, and minimum crossing distances from Sicily exceed 80 km, based on modern bathymetry. Whilst the central Mediterranean is seismically active, there is no direct evidence to suggest substantial tectonic changes to submerged landscapes between Sicily and Malta within this timeframe. High resolution bathymetric studies have revealed paleoshore platforms, shorelines, and channels/river mouths around Malta to a maximum depth of 129–132 m below sea level (35, 36). Examination of the western margin of the land-bridge between Malta and Sicily similarly identified LGM coastline features at 119–131 m below sea level (37). These studies suggest that Malta and Sicily remained connected by a land bridge until sea levels rose to within 100 m of their present level, at which point the connection was severed (36–38). On the basis of the models used here, Malta became isolated from Sicily at some point between ca. 14.5 and 13.8 ka (sea level = −90 m (38)), but most certainly by 12.9 ka (sea level = −75 m (36)), consistent with our analysis. Different models of sea level changes could impact the tempo of land size changes, though these are most likely to push the time frame of Malta's isolation further back in time (31). Our results consequently support the conclusion that Maltese foragers occupied the island when broadly comparable to its current configuration and were not the descendants of a stranded population that survived in isolation for millennia.

The impact of sea level changes on land size is the dominant trend that constrained potential population sizes. Variability in NPP and changing shorelines typically adds stochasticity to this patterning, though for small population sizes, this variability may exacerbate their vulnerability (39). One clear instance in which changes in NPP may have substantially driven population size change is with the Bølling–Allerød interstadial, where increased NPP leads to a pronounced peak in population size without substantial change in land area, immediately before the disconnection of Malta and Sicily. Whilst direct palaeoenvironmental proxy data remains scarce for the earlier Holocene, changes in molluskan fauna (40) and the presence of gypsum deposits indicating high-levels of evaporation (41), associated with the 8.2 ka event may indicate finer-scale climatic events that would have suppressed NPP and posed direct challenges to forager populations.

Using our model, the highest predicted Maltese Mesolithic forager census population sizes that could be supported range between 144 and 170 individuals, expanding on comparable approaches presented by previous researchers (26–29, 42) Our census population size estimates for Malta are close to broadly cited effective population thresholds for maintaining short term (i.e. up to five generations) viability, yet substantially below thresholds for maintaining long-term viability. In the case of the latter, an effective population size of 1,000 individuals, and substantially larger census population size (4) is required. Census population size estimates for Sicily persistently exceed such thresholds, and patterning we identify is broadly reflective of trends evident in studies of ancient DNA (42) supporting the island as likely to sustain a viable and robust forager population. In contrast, the ongoing presence of the Mesolithic on Malta for a millennium or more would have required repeated, long-distance sea crossings. Within these constraints, Malta may have been exploited seasonally as part of a broader forager network, or occupied repeatedly for disparate, extended periods. The inferred population size that Malta could support is consistent with the kind of seasonal-scale foraging groups which have been observed in many ethnographic studies (26, 34) rather than an entire, self-sustaining, population.

Our estimates of population size are based on terrestrial NPP and the proximity to the coast, with the latter offering a possible proxy for supplementing terrestrial NPP with maritime resources. Ordonez and Riede (27) review a range of different parameters to identify which has a limiting impact on population size across terminal Pleistocene and early Holocene Europe, with TAP a key factor in southern Europe. For Malta, and the majority of Sicily, our Miami NPP estimates are consistently TAP-limited, which therefore accounts for the potential role of TAP on our population size estimates. This suggests that models that use ethnographic data that share a precipitation limit on Miami NPP offer more appropriate, lower estimates of population densities in the region. Archaeological evidence suggests that in spite of low species diversity on Malta (23), terrestrial resources played a key role in Mesolithic diets (25). The persistence of fragile endemic deer populations throughout the Mesolithic and into the Neolithic suggests no over-exploitation of this key terrestrial resource, in contrast to what is seen in Sicily (43). This finding is at odds with the potential for a longstanding and viable resident human population on Malta following isolation from Sicily ca. 14.2 ka persisting until archeological identification ca. 8.5 ka. Rather, it is consistent with the limited exploitation of terrestrial resources by relatively small populations.

By focusing explicitly on terrestrial NPP, our model does not account directly for the potential for marine resources to have been used to supplement Mesolithic diets. It is known that Late or “Second” Mesolithic hunter-gatherers had a maritime diet component that varied in different regions from modest to significant (44). In Sicily, Late Mesolithic hunter-gatherers certainly exploited seafood (42). On Malta, besides intertidal rocky shore gastropods, fish and marine mammals only makes up a very small percentage of the Latnija faunal assemblage, which is dominated terrestrial resources such as deer (25). Based on the archaeological evidence, the impact of omitting marine NPP from the population estimates is therefore unlikely to substantively influence our results. Both the appraisal of the distance to the coast and the contribution of fishing to the diet of ethnographic populations used in modeling offer alternate proxies to tackle this issue, with greater proximity to the coast and larger proportions of fishing in forager diets relating to larger population sizes. Whilst contributions from marine resource NPP could plausibly have enabled populations to achieve short-term viability (e.g. 100 years), this appears unfeasible for the scale of population required to maintain long-term viability (e.g. 1,000 years). This is supported by studies of larger Mediterranean islands, such as Cyprus, where an initial colonizing population of ca. 2,700 was argued as a minimum to achieve population viability (13).

The emerging evidence suggests that Malta may have formed part of a larger seagoing network of Mesolithic hunter-fisher-foragers that were capable of sustained, long-distance, sea journeys. The presence of European hunter-gatherer ancestry in the genomes of Tunisian Epipalaeolithic communities supports the idea that these groups may have been capable of traveling even longer distances (45). While these distances invoke the idea of substantial risk—something borne out by experimental journeys of similar distances (46)—the nature of the currents and the sparseness of islands in the south-Central Mediterranean compared to the Aegean, for example, at least facilitates southwards movement across long distances. As a result, the Central Mediterranean appears to have offered its own unique parameters for a seafaring “nursery” (47), in which long-distance voyaging connected islands and continents long before the Classical Era. The Maltese prehistoric archaeological record indicates repeated hiatuses in prehistory, even following the arrival of agro-pastoral societies (48, 49), accentuating both the fragility of human populations living on small islands and the importance of connectivity across the sea. This south-central Mediterranean “sea of islands” (50) supports a reconsideration of the earliest colonization of all small and remote Mediterranean islands, and, at least on Malta, a re-evaluation of the transition from pristine natural systems to human dominated landscapes.

## Materials and methods

A number of studies have been undertaken to examine the relationship between NPP and density of forager populations from ethnographic records (9, 10). Here, we employ the dataset curated by Tallavaara et al. (9), which combines observations from datasets of both Binford (26) and Kelly (34). In order to explore potential population densities on Malta through the terminal Pleistocene and early Holocene, we employed the CHELSA 21k TraCE dataset (32) which provides downscaled palaeoclimatic transient model outputs with ca. 1 km^2^ cells at 100-year time steps spanning the timeframe of interest, accessed using the *pastclim* R package (51). These were cropped to include Malta, Sicily and their immediate environs. We interpolated sea level estimates for the Mediterranean presented by Lambeck et al. (31) to match time steps in the CHELSA 21k TraCE dataset, focused on average values within a two sigma window. We used a bathymetric model (GEBCO (30)), to generate masks for past land surface areas, cropping each time step in the CHELSA TraCE21k dataset to the corresponding sea level estimate.

To establish a relationship between reported NPP and potential forager population size in the past, we calculated NPP using the Miami model. This model was first established by Lieth (33) from empirical datasets, recognizing the critical limiting role that precipitation and temperature play on NPP. Subsequently, a range of further climatic factors has been identified that improve correlation between observed and modeled NPP values, such as evapotranspiration, yet calculation of these factors remains complex. The continued use of the Miami model reflects its reliability to make estimates for NPP based on commonly available and easily calculated climatic parameters: MAT (bio01) and TAP (bio12). We calculated NPP using the Miami model for the modern (0 ka) time slice of the CHELSA 21k Trace dataset, based on 305 forager population locations for which subsistence was purely derived from hunting, gathering and fishing practices, excluding impacts of contact with farmers, which comprises our full dataset. We subset this full dataset to those sites for which precipitation was the limiting factor for Miami NPP values, matching the dominant factor on the Maltese Islands and Sicily (see also Ref. (27)). We then subsetted both these datasets (referred to as T&P for the full dataset, and P for those based on ethnographic data in which precipitation is the limiting factor on Miami NPP) based on the proportional role of fishing within diets (as reported by Binford and Kelly (9, 10)), using thresholds at <50, <25, and <10% to explore the potential impacts of marine contributions to the diet and their influence on potential population sizes.

We undertook preliminary analysis of alternate modeling approaches, including use of linear models, polynomial models, and GAMs, and a focus on the relationship between population density and NPP, as well as between population with NPP and the distance to the coast, the latter providing another means to explore potential influence of access to marine resources. We conducted our analysis using GAMs focusing on the interaction between population density with NPP and the distance to the coast, which explained higher proportions of deviance than other modeling approaches. For the T&P dataset we used thin-plate regression splines for the full dataset and <50% fishing dataset, but the reduced size of the T&P < 25% and <10% datasets required the use of shrinkage thin-plate splines. For the P dataset, use of shrinkage thin-plate splines was used for all models. Whilst in each case thin-plate regression splines resulted in larger proportions of deviance explained, with a maximum of 61.7% for the T&P Full dataset, the use of shrinkage thin-plate splines offsets the risks of overfitting (52) (Table 1).

**Table 1:** GAM model fit statistics (*indicates us of thin-plate regression splines; all other models shrinkage thin plate splines were employed).

Model	Precipitation and temperature (P&T)		Precipitation (P)	
	Deviance explained	*r* ^2^	Deviance explained	*r* ^2^
Full	61.7*	0.605*	45.5	0.449
<50% fishing	57.5*	0.566*	41.1	0.402
<25% fishing	34.8	0.337	31.7	0.304
<10% fishing	24.3	0.218	19.9	0.172

Estimates for NPP were then generated for each cell for each time slice and subsequently converted into an estimate of forager population density per cell, and then the population size estimate per cell was undertaken based on an average cell size of ca. 0.697 km^2^ at 36°N. Population size estimates were then aggregated by discrete landmasses for each time slice to enable examination of the total estimated population for an individual island through time. Here, we focus specifically on the Maltese Islands (Malta, Comino, Gozo) and Sicily, which in the past formed a single, connected landmass, and track changes in predicted forager population sizes over the past 21 thousand years as they become discrete islands, summing population density results for the Maltese Islands. We track the distance between these islands as they separate from one other during the terminal Pleistocene as important ancillary information to examine potential population viability on Malta. Here, we are specifically interested in the extent of sea voyaging required for population contacts. Firstly, we calculated cost path distances between modern Malta and Sicily where no cost was attributed to travel over land, but a cost was incurred to travel over the sea that was consistent with the distance of sea crossing, thus estimating the number of kilometers of sea voyage required to go between the two locations. We then differentiated the total distance of sea crossing required to travel from Malta to Sicily as sea levels fluctuated, as well as the maximum single sea crossing required for instances in which intermediary islands could be used to break the journey.

## Data Availability

All data used in the study comes from cited sources, with analytical code hosted at https://github.com/jblinkhorn/MaltaPopulationDensity.

## Preprints

This manuscript was posted on a preprint: https://osf.io/preprints/socarxiv/5mze4_v1.
